# Paraneoplastic sensorimotor neuropathy and ventral cauda equina nerve root enhancement as initial presentation of small cell lung carcinoma: a case study

**DOI:** 10.1186/s12883-021-02404-4

**Published:** 2021-09-27

**Authors:** Meshari Alsaeed, Chloe A. R. Lim, Alyson Plecash, Tychicus Chen

**Affiliations:** 1 Division of Neurology, Department of Medicine, University of British Columbia, 2775 Laurel Street, Vancouver, BC V5Z 1M9 Canada; 2 Faculty of Medicine, University of British Columbia, Vancouver, BC Canada

**Keywords:** Paraneoplastic neuropathy, Paraneoplastic antibodies, Onconeural antibodies, Small cell carcinoma, Case report

## Abstract

**Background:**

Paraneoplastic neurologic syndromes (PNS) are rare, however, are important to recognize as oftentimes they precede the detection of an occult malignancy. Our case highlights a rare circumstance of paraneoplastic radiculoneuropathy and the importance of recognizing PNS in antibody negative disease, as is the case in up to 16% of sensory neuronopathies, and the process of excluding other etiologies.

**Case presentation:**

We discuss a 51-year-old man who presented with asymmetric subacute sensorimotor deficits in the lower limbs. Initial clinical examination showed weakness throughout the right lower limb and normal strength on the left with objective numbness in a mixed dermatomal and stocking-glove distribution. Electrophysiology was consistent with axonal sensorimotor neuropathy. Cerebrospinal fluid showed pleocytosis and elevated protein. Intravenous immunoglobulin treatment was given with some improvement in pain symptoms but no measurable motor improvement. Following clinical and electrophysiologic deterioration the patient was transferred to a tertiary centre. Magnetic resonance imaging of the spine showed smooth enhancement of the ventral caudal nerve roots. Chest computed tomography revealed left lower vascular scarring. Further positron emission tomography scan imaging identified fluorodeoxyglucose avid right lung lymphadenopathy. Bronchoscopy-guided biopsy revealed small cell lung carcinoma. Onconeural and antiganglioside antibodies were negative. The patient was then transferred to a medical oncology ward where he underwent chemoradiotherapy and subsequently experienced improvement in his motor function, supporting that his neurological condition was indeed secondary to a paraneoplastic process.

**Conclusions:**

Onconeural negative paraneoplastic radiculoneuropathy can precede diagnosis of small cell lung carcinoma. If considered early and adequately investigated, it can allow earlier diagnosis and treatment of underlying malignancy, improving overall and neurological prognosis.

## Background

Paraneoplastic neurologic syndromes (PNS) are rare occurrences present in 0.01% of patients with cancer [1]. Although these syndromes are often associated with positive onconeural antibodies, their pathogenic roles remain largely unknown; additionally, a significant number of patients are seronegative [2–5]. The timing of PNS in relation to malignancy diagnosis is variable. Presentation can parallel or follow the development of a primary cancer, can occur with malignancy relapse after treatment, or can actually precede malignancy diagnosis and even development [6].

PNS can present with a wide clinical spectrum, although specific onconeural antibodies are often linked to specific disease presentations (i.e. SOX-1 is highly associated with malignancy associated Lambert-Eaton Myasthenic Syndrome) [7]. Neuropathy related PNS typically presents with a progressive subacute onset, often with early involvement of the upper extremities and possible CNS involvement (e.g. limbic encephalitis). Often, there are also concurrent constitutional symptoms [7]. Small cell lung carcinoma (SCLC) and thymoma tend to be malignancies most associated with PNS, although multiple cancers have been implicated in various syndromes [6]. Nerve root enhancement secondary to SCLC associated PNS is rare with two reported cases [7, 8]. We herein present a case of SCLC preceded by paraneoplastic sensory and motor radiculoneuropathy with nerve root enhancement.

## Case presentation

In August 2020, a 51-year-old right-handed male non-smoking marathon runner, with no past medical or relevant family history, presented to a community hospital emergency room with a fall following a four-month history of progressive asymmetric numbness and weakness in the legs. This was associated with unexplained 10-pound weight loss. His exam revealed Medical Research Council (MRC) grade 4 strength throughout the right lower limb with normal motor strength in the left lower limb and associated areflexia. Examination of the upper extremities was unremarkable. Investigations were initiated by the family doctor, which consisted of hemoglobin A1c, TSH, and vitamin B12 levels, which were unremarkable. An inpatient work-up including cerebrospinal fluid (CSF) analysis revealed elevated white blood cell (WBC) count and protein of 14 × 10^6^/L and 1.1 g/L respectively. Magnetic Resonance Imaging (MRI) of the spine was also performed and was unremarkable. The working diagnosis was Guillain-Barre Syndrome (GBS), and he was treated with a 5-day course of Intravenous immunoglobulin (IVIg). After discharge, his electromyography (EMG)/nerve conduction study revealed a primarily lower extremity sensorimotor axonal neuropathy with complete sparing of the upper extremities. Right peroneal motor response was absent with reduced right peroneal sensory amplitude and velocity. Needle EMG showed increased insertional activity with fibrillations and positive sharp waves in the right rectus femoris, right adductor longus, and right tibialis anterior, as well as active denervation in the L5 paraspinous muscles.

In October 2020, he was seen by a community neurologist who identified significant worsening in neurological status. Therefore, he was referred to a neuromuscular specialist in a tertiary centre. Clinical exam at this time identified MRC grade 0 hip flexion, grade 0 knee extension, grade 2 knee flexion, and grade 0 ankle dorsiflexion on the right side. Examination of the left side revealed grade 2 hip flexion, grade 2 knee extension, grade 4 knee flexion, and grade 4 ankle dorsiflexion. Repeat electrophysiologic studies again demonstrated a primarily axonal sensorimotor neuropathy. Due to the patient’s significant clinical and electrophysiologic deterioration, he was referred for admission to the neurology service of a tertiary centre for further work-up.

**Fig. 1:**
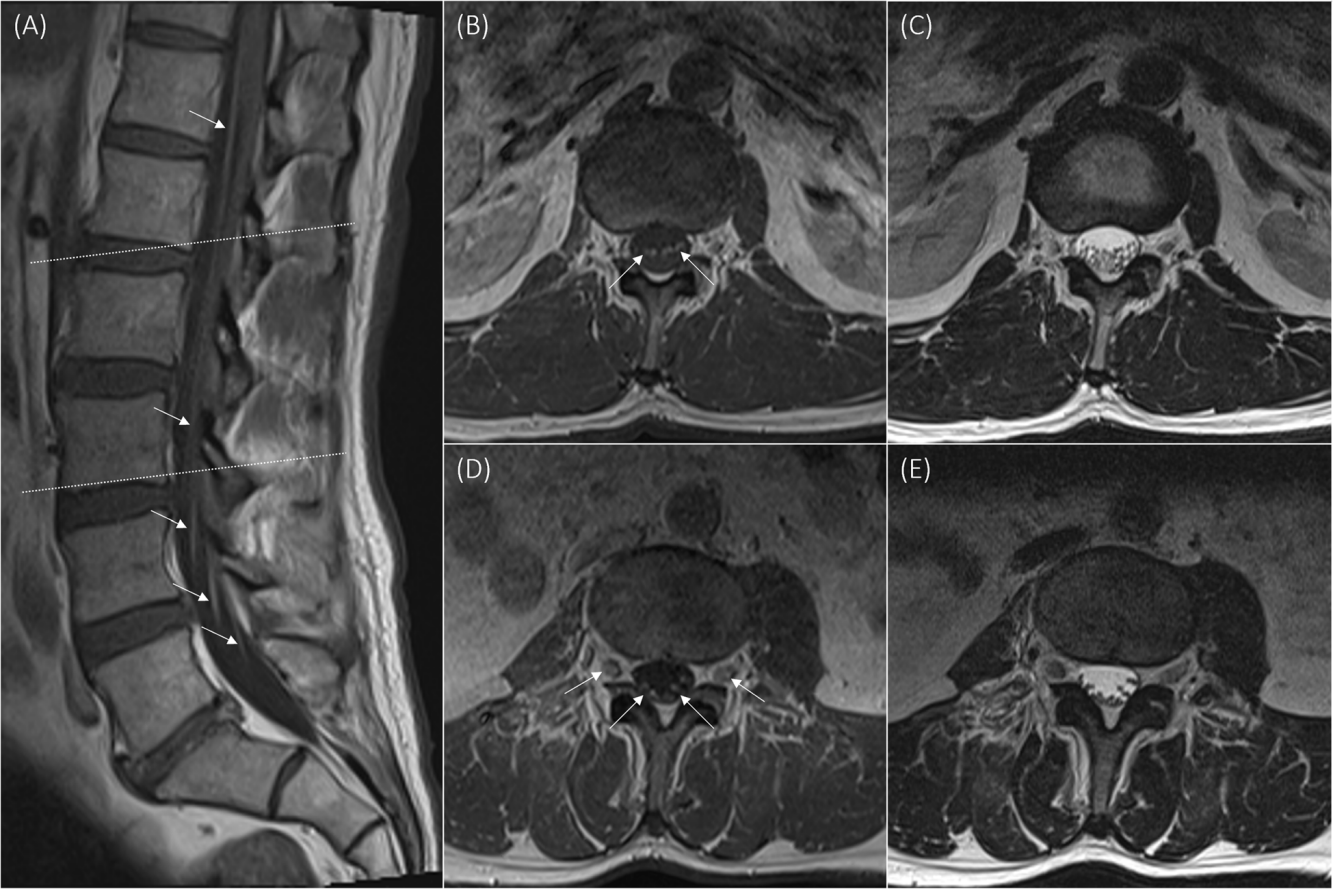
MRI lumbar spine: T1 post-gadolinium sagittal (A) and axial (B, D) views showing smooth enhancement of ventral nerve roots (arrows), with corresponding T2 axial (C, E) views. Panel A, upper dashed line corresponds to axial slices B and C, lower dashed line corresponds to axial slices D and E. MRI = magnetic resonance imaging

A complete spinal MRI with gadolinium showed smooth enhancement of the ventral nerve roots of the cauda equina extending from the conus medullaris ventrally at T11-T12 to the lumbosacral thecal sac (Fig. 1), which can be seen in immune-mediated syndromes such as Guillain-Barre syndrome (GBS) and Chronic Inflammatory Demyelinating Polyneuropathy (CIDP). Denervation atrophy was noted over the right psoas and dorsal paraspinal muscles. Follow-up MRI of the pelvis and lumbosacral plexus again demonstrated a similar enhancement pattern and evidence of mild Short-T1 Inversion Recovery (STIR) high signal and enlargement in extrathecal lumbosacral plexus. Repeat CSF from November 10th and 20th revealed persistently elevated protein (0.58 g/L and 1.67 g/L respectively) and pleocytosis (cell count of 13 × 10^6^/L and 8 × 10^6^/L respectively) with no oligoclonal bands. Flow cytometry was negative twice for neurolymphomatosis. Given the high concern for malignancy, the patient was further investigated for lymphoma with random skin and bone marrow biopsies, which were negative. Left sural nerve biopsy showed mild, nonspecific changes, with patchy loss of larger myelinated fibers, otherwise normal fascicle architecture, occasional scattered endoneurial lymphocytes, and no evidence of vasculitis, amyloid deposition, or neoplastic infiltrates. Gastrocnemius biopsy also showed mild, nonspecific changes, with rare scattered atrophic and regenerating fibers and no inflammatory infiltrates. Serum protein electrophoresis revealed mild polyclonal increase in gamma globulins, likely related to prior treatment with IVIg, without a monoclonal peak. Low-dose full-body Computed Tomography (CT) was also negative. Porphyria screen was negative.

**Fig. 2:**
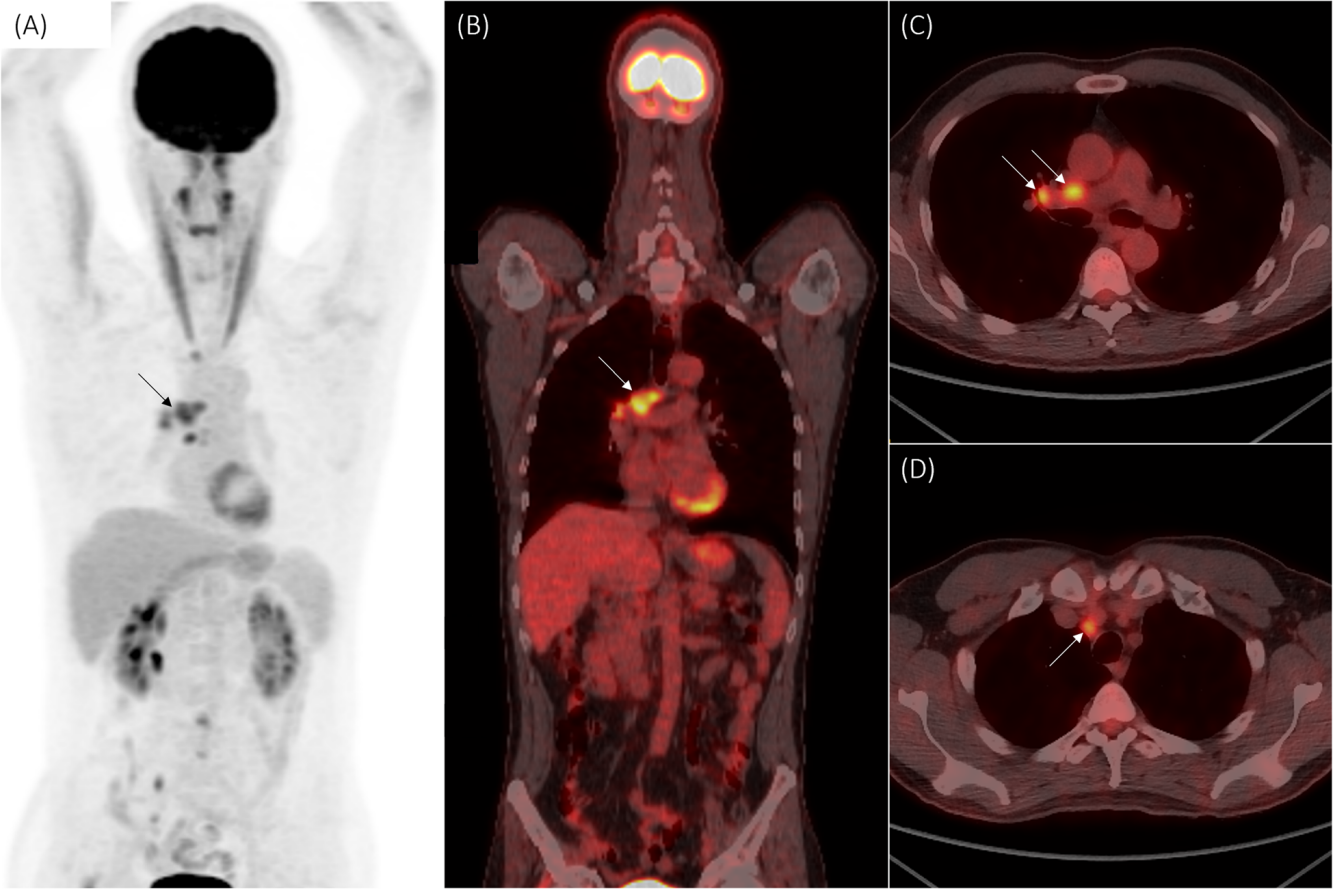
FDG-PET body scan: multiple lymph nodes with FDG-avid uptake in right subcarinal and right hilar (A-C) and superior right paratracheal (D) areas. FDG = fluorodeoxyglucose, PET = positron emission tomography

However, full-dose CT chest, abdomen, and pelvis with contrast revealed vascular scarring of the left lower lobe of the lung and 7 × 6 mm soft tissue nodule in the right upper lobe apex. Subsequent positron emission tomography (PET) identified fluorodeoxyglucose (FDG)-avid right pretracheal, precarinal, subcarinal, and hilar lymphadenopathy (Fig. 2) with no hypermetabolism in the nerve roots. Urgent bronchoscopy and lymph node biopsy was performed, which confirmed the diagnosis of SCLC. Serum and CSF paraneoplastic and peripheral neurologic diseases antibody panels run at Mitogen Advanced Diagnostics Laboratory (Calgary, AB, Canada) returned negative (including onconeural antibodies anti-Hu, anti-Ri, anti-amphiphysin, anti-Yo, anti-Ma, anti-CV2.1, anti-recoverin, anti-SOX1, anti-titin, as well as anti-GM1–3, anti-GD1a-b, anti-GQ1b, anti-GT1b).

In terms of treatment, the patient completed the initial course of IVIg as previously described, as well as a repeat course following his admission to the tertiary center. He was then treated with IV methylprednisolone 1 g daily for 5 days, followed by a slow oral prednisone taper. He was started on trimethoprim-sulfamethoxazole prophylactically prior to the bronchoscopy results. After his diagnosis was confirmed as SCLC, he was transferred to a medical oncology ward at his secondary care centre to receive radical chemoradiotherapy. He went on to have four cycles of carboplatin, etoposide, and radiation therapy (40 Gy in 15 fraction). Two months later, the patient regained ten pounds of weight and is able to independently transfer from his bed to his chair and walk with a walker and ankle-foot orthotic for persistent left foot drop.

## Discussion

This report describes a patient with acute sensorimotor polyradiculoneuropathy with electrophysiologic studies consistent with an axonal process involving the nerve roots and peripheral nerves. All other investigations, including antiganglioside antibodies and vasculitic studies, were negative. The patient was then diagnosed with biopsy proven SCLC. However, all onconeural antibodies tested were negative. Given the absence of any other possible cause following a thorough investigation, the patient’s presentation was most compatible with a seronegative paraneoplastic radiculoneuropathy. This diagnosis is further strengthened by his neurologic improvement following chemoradiotherapy.

An important differential diagnosis to consider is leptomeningeal carcinomatosis - the direct invasion of a tumor to the meninges surrounding the brain and/or spinal cord, causing a variety of neurological symptoms and signs. Leptomeningeal carcinomatosis is most commonly associated with lung cancer, breast cancer, and melanoma, although typically non-small cell lung cancer as opposed to SCLC [9]. It can be diagnosed through CSF analysis, with the typical findings including CSF pleocytosis, elevated protein, and malignant cells found on cytology and flow cytometry. One retrospective study of 113 patients with identified leptomeningeal carcinomatosis found the median CSF white blood cell count to be 33 × 10^6^/L and median protein to be 1.1 g/L [10]. Imaging findings, typically MRI, can be supportive - the most common MRI findings include patchy involvement of the nerve roots as well as occasional matting and intradural extramedullary nodules [11]. FDG-PET scan can also be helpful, with a number of cases with lumbar nerve root leptomeningeal metastases demonstrating increased FDG uptake and hypermetabolism in the lumbar spinal canal [12, 13]. Finally, SCLC with leptomeningeal carcinomatosis portends a poor prognosis, in the order of months, with median survival being 1.6 months [14].

In our case, although leptomeningeal carcinomatosis could not be definitively excluded, as this can only be done with a biopsy of the affected nerve or nerve root with significant morbidity, this was thought to be less likely for a number of reasons. The patient’s lack of significant deterioration more than 6 months following initial diagnosis and one year from onset of symptoms would be atypical. Furthermore, MRI imaging findings were not consistent, given the smooth pattern of enhancement and there was no associated lumbar hypermetabolism identified on PET imaging. In addition, CSF flow cytometry and cytology were negative twice, and the pleocytosis was not very high which may be more in keeping with albuminocytologic dissociation given the much higher relative protein. A diagnosis of paraneoplastic radiculoneuropathy was favoured although no antibody was identified.

Paraneoplastic neurologic syndromes (PNS) are autoimmune diseases which are linked to cancers but not caused by direct tissue invasion. They are typically identified by particular autoantibodies, but this is not always the case. There are multiple types of PNS, and it is useful to categorize them based upon the pathogenesis. The presence of antibodies against intracellular proteins (e.g. Anti-Hu) usually suggests an underlying paraneoplastic process which is typically mediated by cytotoxic T-Cells. These antibodies do not usually have a directly pathogenic role. Conversely, antibodies against synaptic proteins (e.g. Anti-NMDA) can represent a paraneoplastic process, but they can also occur spontaneously. These antibodies are typically directly pathogenic. This difference is critical as it would guide further management, as if the antibody is not directly pathogenic, there is minimal role for immune therapy or plasma exchange [15, 16].

To our knowledge, our patient demonstrates the first reported case of seronegative axonal sensorimotor lumbosacral radiculoneuropathy preceding a diagnosis of SCLC. There is one reported case of bilateral vocal cord paralysis and cervicolumbar radiculoneuropathy preceding a diagnosis of SCLC which was then found to be anti-Hu positive, which unfortunately did not respond well to immunotherapy or chemotherapy [17]. A recent review by Devine et al. demonstrates that paraneoplastic radiculoneuropathies are most often associated with anti-CV2.1 and anti-amphiphysin, with the most commonly associated tumors being breast cancer and SCLC [18]. Further, there have been reported cases of patients with overlapped anti-CV2.1 and anti-amphiphysin developing paraneoplastic radiculoneuropathy associated with SCLC [19]. Patients with anti-amphiphysin alone were noted to show some improvement with immunotherapy. Patients with anti-CV2.1 were noted to be more refractory. In general, there was no association demonstrated between treatment of the malignancy and neurological improvement with either anti-amphiphysin or anti-CV2.1. This lack of improvement with therapy may be due to these antibodies targeting intracellular neuronal proteins [19]. In contrast, our patient’s improvement with malignancy treatment may point to improved prognosis in a suspected seronegative paraneoplastic radiculoneuropathy, although this would require larger studies to establish, and to our knowledge our case represents the first of its kind reported.

According to the literature, the most common cancers associated with axonal sensorimotor neuropathies are SCLC and adenocarcinoma, with the most commonly associated antibodies being anti-Hu and anti-CV2. With respect to demyelinating sensorimotor neuropathies, the most common cancers include lymphoma, adenocarcinoma, and SCLC, with the most commonly associated antibody being anti-CV2 [7].

The clinical features of a paraneoplastic axonal sensorimotor radiculoneuropathy typically resemble those of an axonal variant of GBS/CIDP, with subacutely ascending weakness, as well as ascending sensory loss and sensory loss in a dermatomal distribution. Furthermore, these patients also tend to experience significant neuropathic pain requiring treatment [20]. One can consider Acute Motor and Sensory Axonal Neuropathy (AMSAN), however, this is rarely isolated to the lower extremities [21]. Electrophysiologically, nerve conduction studies characteristically show axonal fiber loss while EMG shows active denervation in affected muscles, including paraspinal muscles [7, 21]. In terms of imaging studies, these are often normal, but can also show smooth enhancement of the nerves, as seen in our case which also demonstrated mild STIR high signal [7, 8].

Patients with seronegative paraneoplastic disorders are frequently underdiagnosed [2]. Given that the neurological symptoms often precede the cancer diagnosis, prompt identification of a paraneoplastic process can lead to earlier cancer detection, leading to earlier therapy and improved outcomes [6]. Therefore, a thorough diagnostic approach is necessary.

On initial encounter with the patient, it is important not only to delineate the history of the neurological process, but also to assess for cancer risk factors, such as weight loss or a significant family history of malignancy, as these may guide further investigations. Beyond this, in a patient with a subacute and progressive neurological disorder with no clear cause, a paraneoplastic syndrome should be considered and antibodies should be sent. In a patient with positive antibodies, a thorough search for the associated cancer should be undertaken. In patients with no positive antibodies, a multi-pronged approach becomes necessary, wherein a search for an alternative diagnosis (e.g. infection, vasculitis, sarcoidosis, etc.) is done alongside cancer screening investigations. An empiric trial of immunotherapy can also be beneficial, although it may make diagnosis more difficult. Extensive imaging is typically required in these cases. Positron emission tomography is also helpful and can provide biopsy targets which was crucial in this case. Following identification of the malignancy, appropriate treatment can be undertaken. In patients where no malignancy can be identified and no other cause has been found, repeat antibody testing may be helpful. It is also imperative that the patient is up to date with their age appropriate malignancy screening [22].

In conclusion, our patient presented with an acute sensorimotor radiculoneuropathy with subsequent diagnosis of a SCLC with no positive antibodies. It is suggested that in a patient presenting this way with no clearly identifiable cause, there should be no delay in thorough malignancy screening, as it could aid in early cancer diagnosis and prompt treatment, thereby improving prognosis.

## Data Availability

Data sharing not applicable to this article as no datasets were generated or analysed during the current study.

## Abbreviations

AMSAN: Acute motor and sensory axonal neuropathy
CIDP: Chronic inflammatory demyelinating polyneuropathy
CNS: Central nervous system
CSF: Cerebrospinal fluid
CT: Computed tomography
EMG: Electromyography
FDG: Fluorodeoxyglucose
GBS: Guillain-Barre syndrome
IVIg: Intravenous immunoglobulin
MRC: Medical research council
MRI: Magnetic resonance imaging
PET: Positron emission tomography
PNS: Paraneoplastic neurologic syndromes
SCLC: Small cell lung carcinoma
STIR: Short-T1 inversion recovery
TSH: Thyroid stimulating hormone
